# Next generation multiplexing for digital PCR using a novel melt-based hairpin probe design

**DOI:** 10.3389/fgene.2023.1272964

**Published:** 2023-11-10

**Authors:** Rebecca L. Edwards, Johanna E. Takach, Michael J. McAndrew, Jondavid Menteer, Rachel M. Lestz, Douglas Whitman, Lee Ann Baxter-Lowe

**Affiliations:** ^1^ Department of Pathology and Laboratory Medicine, Children’s Hospital Los Angeles, Los Angeles, CA, United States; ^2^ Luminex Corporation, A Diasorin Company, Austin, TX, United States; ^3^ Keck School of Medicine, University of Southern California, Los Angeles, CA, United States; ^4^ Division of Cardiology, Children’s Hospital Los Angeles, Los Angeles, CA, United States; ^5^ Division of Nephrology, Children’s Hospital Los Angeles, Los Angeles, CA, United States

**Keywords:** absolute quantification, digital PCR, higher-order multiplexing, mdPCR assay, melt-based hairpin probe design

## Abstract

Digital PCR (dPCR) is a powerful tool for research and diagnostic applications that require absolute quantification of target molecules or detection of rare events, but the number of nucleic acid targets that can be distinguished within an assay has limited its usefulness. For most dPCR systems, one target is detected per optical channel and the total number of targets is limited by the number of optical channels on the platform. Higher-order multiplexing has the potential to dramatically increase the usefulness of dPCR, especially in scenarios with limited sample. Other potential benefits of multiplexing include lower cost, additional information generated by more probes, and higher throughput. To address this unmet need, we developed a novel melt-based hairpin probe design to provide a robust option for multiplexing digital PCR. A prototype multiplex digital PCR (mdPCR) assay using three melt-based hairpin probes per optical channel in a 16-well microfluidic digital PCR platform accurately distinguished and quantified 12 nucleic acid targets per well. For samples with 10,000 human genome equivalents, the probe-specific ranges for limit of blank were 0.00%–0.13%, and those for analytical limit of detection were 0.00%–0.20%. Inter-laboratory reproducibility was excellent (*r* ^2^ = 0.997). Importantly, this novel melt-based hairpin probe design has potential to achieve multiplexing beyond the 12 targets/well of this prototype assay. This easy-to-use mdPCR technology with excellent performance characteristics has the potential to revolutionize the use of digital PCR in research and diagnostic settings.

## 1 Introduction

Digital PCR (dPCR) is used in a wide range of bioscience applications because it offers several advantages over alternative methods for detecting specific nucleic acid targets. One of the major advantages is that dPCR provides absolute quantification of nucleic acid targets without a calibration curve (Doi et al., 2015; Dueck et al., 2019; Peng et al., 2020; Sugimoto et al., 2021; Clausen et al., 2023). Another major advantage is that accuracy and precision of dPCR methods have been reported to be superior to other PCR methods in part because they mitigate factors that affect PCR efficiency (Simmonds et al., 1990; Sykes et al., 1992; Vogelstein and Kinzler, 1999; Hindson et al., 2013; Svec et al., 2015). For situations that require quantifying low-abundance targets present in a complex nucleic acid background, dPCR has become the method of choice (Hayden et al., 2013; Hindson et al., 2013; Doi et al., 2015; Taylor et al., 2017; Lancikova and Hricova, 2020). These improvements over NGS and other PCR approaches are attributed to the fact that in dPCR, samples are partitioned into 10^3^–10^6^ reactions, preferably with each reaction containing zero or one target sequence (Hindson et al., 2011; Madic et al., 2016; Dueck et al., 2019). After amplification, target-positive or target-negative partitions are usually assigned using signals from fluorescent probes. The number of copies of a probe’s target sequence is calculated using Poisson statistics with end-point measurements that provide nucleic acid quantitation independent of reaction efficiency (Dube et al., 2008). However, a major limitation of dPCR is that a single target is typically detected per optical channel and approaches to overcome this (e.g., dividing sample across multiple wells) can be cumbersome.

Several approaches have been explored to increase the multiplexing capacity of dPCR but all have at least one major drawback such as multi-step workflows, extensive cycling, difficulty distinguishing positive and negative results (thresholding), background fluorescence, off-target amplification, and/or amplification bias. For example, a barcode bead-based multiplexed droplet digital PCR method (BB-ddPCR) method can distinguish five viral targets in a single tube but this method requires multiple steps, including pre-amplification of sample material, hybridization of pre-amplified products to beads, bead partitioning, digital PCR amplification of products via the Naica Crystal Digital PCR system (Stilla Technologies, Villejuif, France) and image analysis (Gu et al., 2022). An alternative multiplexing approach for dPCR involves varying the relative concentrations of different probes (i.e., TaqMan) labeled with the same fluorescent dye. In this method each probe is distinguished by a unique endpoint amplitude (fluorescence intensity) within the same optical channel (Zhong et al., 2011; Rajagopal et al., 2019; Jacky et al., 2021a). This approach, recently termed Virtual-Partition digital PCR (VPdPCR) has been reported to detect up to 10 targets per optical channel. In contrast to dPCR which employs a single threshold to separate positive and negative partitions, VPdPCR requires multiple thresholds to identify the number of unique targets present (Jacky et al., 2021b). This method suffers from challenging assay optimization and complex data interpretation. Another approach for multiplex dPCR, universal Digital High-Resolution Melt (U-dHRM) utilizes a high-content U-dHRM chip for microfluidic reaction partitioning fluorescence but this relies on machine learning to match melt curves to a training database (Fraley et al., 2013; Velez et al., 2017).

To overcome the limitations of current multiplex digital PCR methods, take full advantage of dPCR approach, and expand its usefulness, we developed a novel discrete melt-based hairpin probe chemistry. Herein we demonstrate the effectiveness of this multiplex quantification system utilizing a prototype assay which simultaneously distinguishes and quantifies six biallelic loci (12 targets total) in each well. The specificity, sensitivity, ease of use, and reproducibility across analytical samples of varying complexity described here demonstrate the effectiveness of this robust approach. Importantly, this approach has the capacity to increase the number of targets distinguished and quantified in each well beyond that of this prototype assay. This novel melt probe-based approach for digital PCR (mdPCR) presents an important step toward the next-generation of dPCR assays.

## 2 Materials and methods

### 2.1 Primers and probes

**FIGURE 1:**
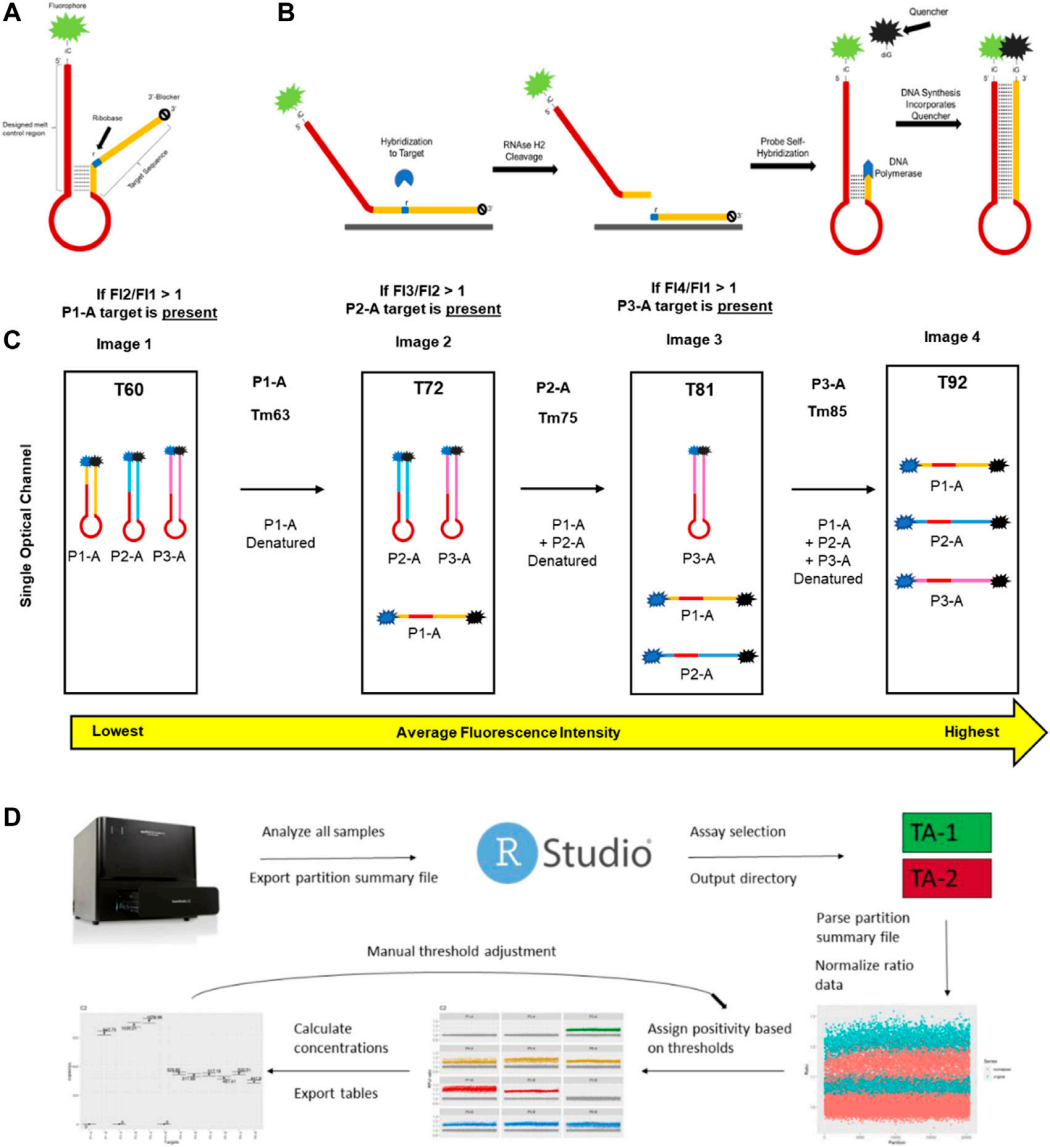
Key aspects of mdPCR. **(A)** Melt-based probe design. **(B)** The probe binds to its amplicon target, causing the formation of an RNA:DNA duplex at the ribobase (r) present in the probe. RNAse H2 present in the reaction mixture cleaves the probe, allowing the 5′ portion of the cleaved probe to form a hairpin which is extended by DNA polymerase. During extension, the DNA polymerase directs a site-specific incorporation of a fluorescent quencher to form a temperature-reversible quenched hairpin. **(C)** Post-amplification, probes containing the same fluorophore can be discriminated based on the hybridization temperature of the final hairpin as determined by discrete melt analysis in which the fluorescence is measured by imaging at four temperatures and the ratios of each set of temperatures (T2/T1, T3/T2, and T4/T3) are used to determine presence/absence of each target. **(D)** Workflow for Erebos data analysis. Optimized positivity thresholds (ratios) for each probe (previously determined experimentally) used were: P1-A > 1.075, P1-B > 1.1, P2-A > 1.06, P2-B > 1.11, P3-A > 1.08, P3-B > 1.12, P4-A > 1.07, P4-B > 1.1, P5-A > 1.085, P5-B > 1.1, P6-A > 1.08 and P6-B 1.075. The copies per μL, total copies per assay input, allele frequency, and 95% Poisson confidence intervals were calculated for each target.

The human genome (GRCh38. p12) was screened to identify biallelic loci with short (2-3 bases) indel sequences that have allele frequencies between 0.4 and 0.6 (Fairley et al., 2020). Sequences from selected loci were evaluated with Primer3 to identify primer sequences flanking the indel regions (Koressaar and Remm, 2007; Untergasser et al., 2012). Novel allele-specific partial hairpin probes were designed to accurately detect three distinct genomic DNA targets in a single optical channel. Each probe is a single-stranded DNA oligo with a 5′-fluorophore adjacent to an isoC followed by a melt-determination region, a partial hairpin region, and a 3′-target specific region that contains a single ribobase (Figure 1). The target-specific region is designed to have more affinity for the amplicon target than the partial hairpin structure at the PCR annealing temperature. Each probe’s hairpin structures have a minimum of six nucleotide bases and a hybridization temperature that is 3°C–4°C lower than the annealing temperature, the Tm of the target-specific region and its amplicon target, but high enough that it is thermodynamically favorable to hybridize during the annealing temperature of the reaction. In the presence of an amplicon target the probe will unfold and hybridize its complementary sequence, allowing RNAseH2 to bind and cleave the RNA:DNA duplex. After cleavage, the hairpin portion of the probe is able to reform and allows DNA polymerase to bind and extend the hairpin using the melt-determination region as a template. Probe fluorescence is dampened by the insertion of an isoG-quencher across from the isoC, creating a temperature-dependent full hairpin sequence. Multiplexing is made possible by utilizing different fluorophores and final hairpin hybridization temperatures. The RNAseH2 activity for each probe was assessed in bulk PCR by running the reaction with a high concentration of target (approximately 10,000 copies of gblock target) to drive the probe signal to plateau. The maximum ∆RFU was assessed for each probe, and probes within the same fluorescent channel were normalized to a final concentration that resulted in the same fluorescent change at PCR endpoint in digital PCR.

This probe chemistry was used to create a prototype mdPCR assay with 12 probes targeting six loci and used to differentiate and quantify alleles with small nucleotide duplications or deletions across four optical channels (Supplementary Table S1). Primers and probes were initially assessed for target specificity using allele-specific short double-stranded DNA fragments (gblocks) to confirm probe specificity. For each locus, probe specificity was assessed by amplification of allele-specific short double-stranded DNA fragments (gblocks) in real-time PCR followed by melt analysis. For each probe, 25 uL reactions containing 1X Multiplex Mastermix (Luminex Corporation, A Diasorin Company), allele-specific primers (excess primer: 400 nM and limiting primer: 100 nM) probe (50 nM) were mixed with approximately 10,000 copies of allele A, allele B, or a no-template control (NTC). Reactions were run in an ABI 7500 Fast Real-Time PCR system (Thermo Fisher Scientific, Waltham, Massachusetts, United States) with the following protocol: 95°C hot start for 2 min 20 s, 60 cycles of amplification (95°C for 10 s, 58°C for 30 s), and then a hold-and-step melt protocol (0.5°C increments from 60°C to 95°C). Multicomponent data was exported and analyzed in custom software to evaluate probe quenching kinetics and final hairpin melt temperatures. Probes were successful if the probe fluorescence decreased in presence of its intended target with a Ct of 25–31 and resulted in a Tm within 2°C of its intended melt temperature, had no significant melt signature with its non-target sequence, and no melt signal in the NTC. Once all twelve probes were selected, the final multiplex assay probe concentrations were normalized so that each probe with the same fluorophore had similar melt deflection (∆RFU) at endpoint.

Successful assays were then tested with human genomic DNA samples to confirm design robustness and multiplex compatibility.

### 2.2 Genomic DNA samples

Samples were prepared from human genomic DNA (hgDNA) from single-source whole blood samples (BioIVT, Westbury, New York, United States) using a Qiagen QIAmp DNA extraction kit (Qiagen, German Town, Maryland, United States). The hgDNA was sheared to approximately 6 kB using g-Tubes (Covaris, Woburn, Massachusetts, United States), quantified by fluorometry using the dsDNA HS (High Sensitivity) Assay (Invitrogen, Thermo Fisher Scientific, Waltham, Massachusetts, United States), diluted to approximately 10,000 human genome equivalents per microliter (hge/μL) and stored at −20 °C prior to use. Assays were confirmed as functional if the locus concentrations were consistent across both fluorometry and dPCR measurement. To assess assay performance, four hgDNA samples (ID 2581, 317, 319 and 592) that had opposing genotypes for the bi-allelic targets in this prototype assay were diluted to approximately 2,500 hge/μL and used to create 50% vol:vol mixtures of two individual’s genomic DNA with minor fractions of 0%, 0.3%, 0.5%, 1%, 2%, 5%, 10%, 25% and 50%.

### 2.3 Multiplex digital PCR

For multiplex dPCR, each 10 µL reaction contained 1X Multiplex dPCR Master Mix (Luminex Corporation, A Diasorin Company) which includes asymmetric primers (excess primer: 400 nM and limiting primer: 100 nM), 200 nM of each normalized probe (Supplementary Datasheet S1), and 4 μL DNA. Each mixture (9 μL) was loaded into the well of a MAP16 digital PCR plate (Thermo Fisher Scientific, San Jose, CA) and covered with 18 μL of Isolation buffer (Thermo Fisher Scientific, San Jose, CA). Each column of wells on the plate was then covered with a strip gasket, and the plates were transferred into a Combinati Absolute Q digital PCR system (currently marketed as Thermo Fisher Scientific Absolute Q digital PCR system, Thermo Fisher Scientific, San Jose, CA) (Dueck et al., 2019; Jiang et al., 2020). Each plate was run with the following protocol: Pressurization at 75 psi for 30 min, 50 psi for 5 min to digitize the sample at room temperature, followed by continued 50 psi pressurization throughout enzyme activation at 96 °C for 10 min, thermal cycling (49 cycles of 93°C for 5 s and 60°C for 20 s), followed by a probe extension cycle with a 76°C hold for 5 s and a 60°C hold for 320 s with images captured at 60°C, 72°C, 81°C, and 92°C**.** Images were taken in four optical channels (blue, green, yellow, and red) to detect probes labeled FAM, AP525, AP559, and AP593, respectively.

### 2.4 Data analysis

For each plate, the raw data for each sample were analyzed using the prototype software 10.15.1 (provided kindly by Combinati). This software identified each of the 20,480 partitions in an image and measured the fluorescence in each partition. The data obtained from the AP593 channel was used to assess the data quality for each reaction. The mdPCR data were exported as a partition summary table in. csv format. The partition summary table contained raw fluorescence information for each partition at all analyzed channels at each temperature (T_1_—T_4_).

After export, these data were analyzed using a proprietary R-based analysis package, termed Erebos (Luminex Corporation, available upon request). In brief, ratio values were calculated for each partition per channel per melt window which correlates to an allele-specific probe. For example, the value of a given partition in the FAM low window would be calculated as: ratio_partition_ = FAM_T2_/FAM_T1_. For each channel/temperature window, partitions with ratios equal to or less than 1.0 were preliminarily assessed as target negative, because the probe fluorescence measurement has limited change between the two temperatures used for detection. The ratio values were then normalized using a rolling window approach such that the average value of a negative partition was ∼1. The positivity of each partition for each probe was determined experimentally, and consistent thresholds were used across all reactions. For each reaction, the aggregate data was collected for each well and used to calculate the quantities of each target present in the sample using the Poisson distribution (Huggett, 2020).

### 2.5 Limit of blank and limit of detection

Human genomic samples were genotyped for target alleles (insertion/deletion alleles aka indels) to select samples that were homozygous for one or more alleles. Four hgDNA samples (ID 2581, 317, 319 and 592) that had opposing homozygous genotypes for their locus were used to determine LoB and LoD for each probe. Dilutions of DNA were used to test approximately 1,000, 5,000, and 10,000 hge/reaction. The LoB for each probe was determined using 16 reactions at each DNA concentration when the target allele was absent as defined by the digital MIQE (Huggett et al., 2015). The theoretical limit of detection was calculated as 3x the standard deviation of the LoB. The LoB was calculated for each probe as follows,

$$\text{LoB}\left(\text{partitions}\right)=\text{mean}\left(\text{false positive partitions}\right)+1.645\left(\text{SD false positive partitions}\right)$$

The value 1.645 represents range of SD from mean to the 95th percentile of a normal distribution.

LoB frequencies (%) were calculated as,

$$\text{LoB frequency}=\frac{\text{LoB}\left(\text{partitions}\right)}{\text{total}\;\text{number}\;\text{of}\;\text{copies}\;\text{per}\;\text{sample}}*100$$

For each of the 12 probes, analytical limit of detection (LoD) is calculated based on LoB (partitions) and the anticipated distribution of results from low-concentration samples.

$$\text{LoD}\left(\text{analytical}\right)=\text{LoB}\left(\text{partitions}\right)+1.645\left(\text{SD false positive partitions}\right)$$

The value 1.645 again representing the 95th percentile of a normal distribution.

LoD frequencies were calculated as

$$\text{LoD frequency}=\frac{\text{LoD}\left(\text{analytical}\right)}{\text{total}\;\text{number}\;\text{of}\;\text{copies}\;\text{per}\;\text{sample}}*100$$

### 2.6 Performance characteristics

Mixtures of genomic DNA from two individuals were prepared to produce minor fractions of 0%–50%. Total assay DNA input was approximately 10,000 hge (33.3 ng DNA). Four replicates of each sample were tested. To determine inter-laboratory reproducibility, human genomic DNA mixtures with minor fractions ranging from 0.3% to 50% were prepared and tested on-site at CHLA. Remaining samples were shipped on dry ice to Luminex laboratories (Austin, Texas) where they were tested and analyzed independently. Each sample was tested 4 times at both sites.

### 2.7 Statistical analysis

Standard deviation (SD), coefficient of variation (CV), and 95% confidence intervals were calculated for samples containing hgDNA mixtures. Assay precision was evaluated by CV% (calculated in Microsoft Excel 365). Linear regression analysis performed using the ggpmisc package in R (v 4.2.1) was used to evaluate inter-laboratory reproducibly.

## 3 Results

### 3.1 Probe mechanism

To address the limitations of existing multiplex digital PCR technology, a novel partial hairpin probe chemistry was developed and assessed for functionality with a melt-compatible digital PCR platform. The key features of the probe design include 1) a target binding region that contains a single ribobase which allows cleavage by RNAseH2 when the probe is hybridized to its DNA target 2) an isoC base adjacent to a fluorophore at the 5′ end of the target binding region, 3) a polyA loop region, and 4) a melt motif sequence which determines the temperature for denaturation of the hairpin loop (Figure 1A). In the absence of an amplicon containing the specific target sequence, the probe self-hybridizes to form a partial hairpin and is fluorescent at all temperatures. When the probe is hybridized to its target, RNAseH2 cleaves at the ribobase and DNA polymerase extends the 3′ region using the 5′ sequence of the probe as the template. The IsoC at the end of the sequence results in incorporation of a dabcyl-labeled isoG base which quenches the 5’ fluorophore of the probe (Figure 1B).

Once extended, the melt determining region of each probe transitions from closed to open between two specific temperatures. Each probe has a unique combination of fluorophore and melt window, yielding precise detection of each target in each partition. Images taken in four optical channels (blue, green, yellow, and red) of a microfluidic dPCR platform at 60°C, 72°C, 81°C, and 92 °C allow specific detection of fluorescence of each probe hybridized to its target sequence (Figure 1C and Supplementary Table S1). In contrast to *TaqMan* probes, which increase fluorescence in the presence of target sequence, these probes decrease fluorescence in the presence of target sequence. One of the advantages of this approach is that the baseline fluorescence for each probe is the fluorescence of the probe below its melt temperature rather than a single baseline for all probes. In the absence of target, the fluorescent signal remains stable at all temperatures. For mdPCR, positive signals are identified as a ratio of the fluorescent signal of the denatured probe to the hairpin probe. An overview of the data analysis pipeline is shown in Figure 1D. Our results demonstrate that this novel melt-based hairpin probe design can achieve specific detection of three genomic DNA targets in each of four optical channels.

### 3.2 Performance characteristics

**FIGURE 2:**
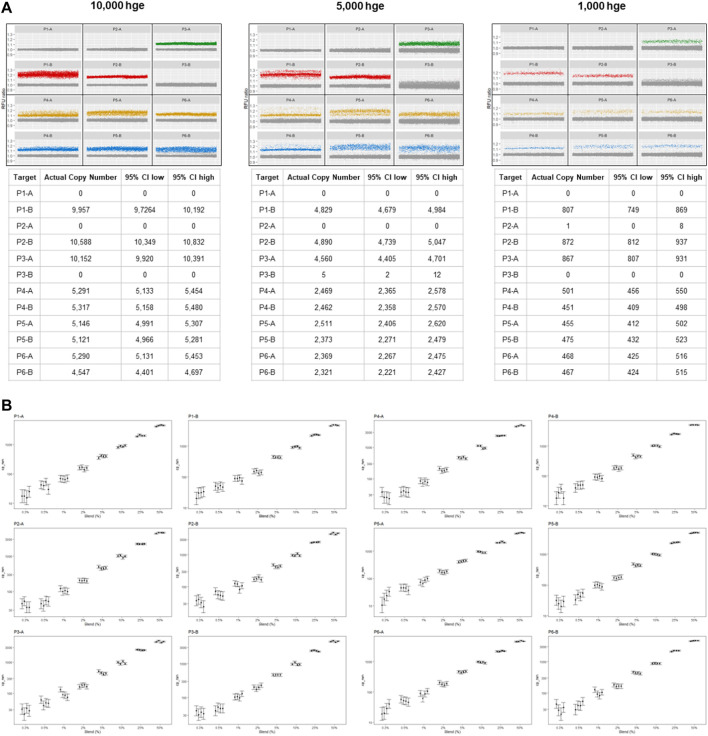
mdPCR assay results. **(A)** Representative data plots and tables from reactions containing 10,000, 5,000, and 1,000 human genomic equivalents from a single individual. Within each partition, 12 targets were simultaneously detected, and Poisson statistics were used to quantify each target in each reaction. For each locus (P1—P6) there were two alleles which were designated **(A, B)**. Partitions below a pre-determined positive RFU ratio were considered to have no target and are shown as gray points which are near 1.0. Partitions above a pre-determined positive RFU ratio were defined as positive and have a different color for each channel (green, red, yellow, blue). **(B)** mdPCR assay results for 12 targets, minor fraction 0.3%–50%. The graphs (on log10 scales) show the number of copies for each minor allele that was detected for each probe (P1-A—P6-B). The results ranged from 30 to 5,000 copies for each of the four reactions. These experiments were carried out using mixtures of two human genomic DNA samples containing minor fractions of each allele at 0%, 0.3%, 0.5%, 1%, 2%, 5%, 10%, 25% or 50%. Four replicates of each sample were tested. DNA input was 10, 000 hge (33.3 ng of DNA). Error bars represent the 95% confidence interval. The CVs for these measurements are provided in Supplementary Datasheet S2.

For this prototype assay, setup for up to 16 samples was completed in 30–60 min and PCR required 2–2.5 h. Human genomic DNA samples were used to demonstrate that 12 probes detecting biallelic indels in six loci can be quantified simultaneously and that each probe is allele specific. For each probe, LoB and LoD were determined using 10,000, 5,000, or 1,000 hge from four individuals (ID 2581, 317, 319 and 592). Figure 2A shows representative results from these experiments**.** Homozygous loci (1–4/sample) were used to measure the false positive rate in 16 replicates for each allele. For an assay input of 10,000 hge and 5,000 hge, the number of false positive partitions was probe dependent and ranged between 0–13 partitions per well. When the assay input was reduced to 1,000 hge, 0–5 false positives partitions were observed. For an assay input of 10,000 hge, LoB frequencies for each probe ranged between 0% and 0.13%. For assay inputs of 5,000 hge and 1,000 hge, LoB frequencies ranged between 0% - 0.26% and 0%–0.62% respectively.

For each of the 12 probes, analytical LoD was calculated based on LoB (partitions) and the anticipated distribution of results from low-concentration samples. For 10,000 hge and 5,000 hge, the analytical LoD was 0–20 partitions and with 1,000 hge the analytical LoD it was reduced to 0–8 partitions. For 10,000 hge, LoD frequencies for each probe were 0%–0.20%. When decreasing the assay input amount to 5,000 hge and 1,000 hge, LoB frequencies were 0%–0.41% and 0% - 0.98% respectively. Results are summarized in Table 1. Figure 2B and Supplementary Datasheet S2, show the assay can accurately and reproducibly quantify all 12 DNA targets in fractions down to 0.3%.

**TABLE 1:** Limit of blank and limit of detection.

Input DNA		P1-A	P1-B	P2-A	P2-B	P3-A	P3-B	P4-A	P4-B	P5-A	P5-B	P6-A	P6-B
**10,000 hge**	LoB	0.40	0.00	5.79	2.23	6.47	4.67	0.67	0.45	0.83	1.47	2.11	12.96
	LoB Freq	0.00%	0.00%	0.06%	0.02%	0.06%	0.05%	0.01%	0.00%	0.01%	0.01%	0.02%	0.13%
	LoD Theo	0.75	0.00	9.66	3.64	10.65	8.14	1.21	0.83	1.47	2.46	3.48	19.56
	LoD Freq	0.01%	0.00%	0.10%	0.04%	0.11%	0.08%	0.01%	0.01%	0.01%	0.02%	0.03%	0.20%
**5,000 hge**	LoB	0.46	0.00	2.22	3.34	2.99	13.25	0.00	1.77	0.83	3.03	1.27	12.09
	LoB Freq	0.01%	0.00%	0.04%	0.06%	0.05%	0.26%	0.00%	0.03%	0.02%	0.06%	0.02%	0.22%
	LoD Theo	0.86	0.00	3.90	5.18	4.84	20.76	0.00	2.97	1.47	5.14	2.23	19.54
	LoD Freq	0.02%	0.00%	0.07%	0.10%	0.09%	0.41%	0.00%	0.05%	0.03%	0.10%	0.04%	0.36%
**1,000 hge**	LoB	0.00	0.00	0.46	1.91	3.79	5.73	0.00	1.66	1.48	2.85	3.86	4.79
	LoB Freq	0.00%	0.00%	0.05%	0.18%	0.34%	0.62%	0.00%	0.15%	0.15%	0.26%	0.39%	0.43%
	LoD Theo	0.00	0.00	0.86	3.19	6.43	9.01	0.00	2.83	2.50	4.64	6.38	7.77
	LoD Freq	0.00%	0.00%	0.09%	0.30%	0.58%	0.98%	0.00%	0.25%	0.25%	0.43%	0.64%	0.70%

Freq, frequency; hge, human genome equivalent; LoB, limit of blank; LoD, limit of detection; Theo, theoretical.

**FIGURE 3:**
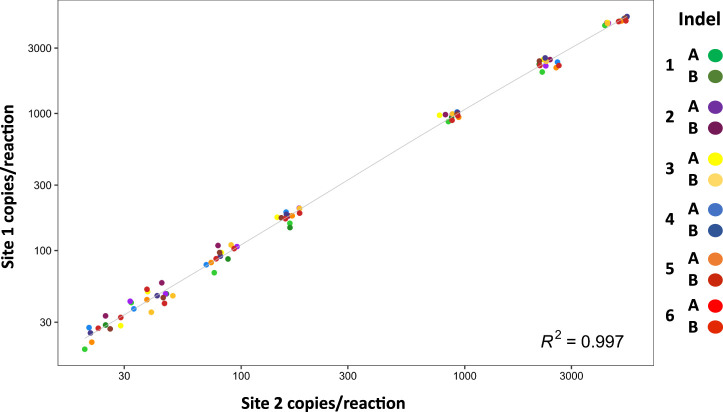
Inter-laboratory reproducibility. The graph shows absolute values of DNA minor fraction concentrations ranging from 30 to 5,000 copies per reaction (*n* = 4) measured at site 1 and site 2. Data are shown on log10 scales. Correlation between site 1 and site 2 is *r* = 0.997.

Inter-laboratory reproducibility was evaluated at two independent sites. Using samples with approximately 10,000 hge, the minor fractions ranged from 0.3%–50% and showed high concordance (Figure 3).

## 4 Discussion

This investigation provides proof-of-concept that melt-based hairpin probes can substantially improve the capabilities of dPCR by achieving robust higher-order multiplexing while maintaining high sensitivity and precision. In contrast to other approaches for multiplexing dPCR assays, this novel melt-probe method was easy to use, fast, and reliable. Using four of the five optical channels on a melt-compatible microfluidic PCR system, the prototype mdPCR assay accurately quantified three nucleic acid targets in a single-optical channel and 12 targets per well. Theoretically, the multiplexing capacity could be substantially expanded by increasing the number of probes in each optical channel and/or using more optical channels.

When mixtures of genomic DNA from two individuals were mixed (0.3%–50% minor fraction), the mdPCR system demonstrated excellent accuracy and precision (Figure 2A). For 10,000 human genome equivalents (33.3 ng), LoB ranged from 0.0% to 0.13% depending upon the probe. Three probes demonstrated LoB of 0.0% and the highest LoB was still acceptable (P6, LoB 0.13%). Given the exceptional performance of other probes, it is likely that LoB for P6 and other sub-optimal probes could be improved by optimizing the probe design. For the conditions tested in these experiments, analytical LoDs were 0%–0.2%. Overall, the assay had a low false positive rate and could reliably detect low levels of target nucleic acids in human genomic DNA samples.

Another feature of this mdPCR system is flexibility to balance throughput with number of discrete targets. For the prototype assay, 12 discrete targets were detected in a single well and up to 16 samples could be tested on a single plate. Probes could be designed to allow detection of many more targets/well. For applications requiring a larger number of probes, several wells could be used for each sample. For example, it might be possible to test 100 targets by using 25 probes per well and four wells/sample. However, it is important to note that increasing the number of probes per channel may also increase the likelihood of probe interactions and affect the overall performance of the assay. To develop higher multi-plexed assays, it is expected that optimization and validation would be extensive.

Only a few reports have described methods for multiplex digital PCR, and these publications describe long turn-around-times, complicated workflows, and complex data analysis (Supplementary Table S2). For example, BB-ddPCR has a lengthy multiple-step workflow, requires a fluorescent microscope for imaging and successful results have only been demonstrated for 5-plex assays (Gu et al., 2022). U-dHRM requires a specialized microchip, considerable time for PCR (e.g., 70 cycles) and a large and comprehensive database of temperature calibrated melt curves for accurate identification of unknown samples (Fraley et al., 2013). A virtual partition PCR approach using TaqMan probes detects 10 probes per optical channel, but this approach requires a complicated setup, has limited sensitivity (5% fetal/aneuploid fraction) and the quantitative accuracy has not been established (Jacky et al., 2021a). The mdPCR method presented here is an exciting approach for expanding the multiplexing capacity of dPCR that overcomes the limitations of other published approaches (Fraley et al., 2013; Jacky et al., 2021b; Gu et al., 2022).

With further optimization for specific targets, and/or sensitivity requirements, this mdPCR system provides a major advance for numerous clinical and research applications that would benefit from 1) accurate and sensitive multi-target detection, 2) rare target quantification, 3) ability to test extremely small DNA samples, 4) and/or rapid turnaround time. Results were reproducible in two independent laboratories (Figure 3) suggesting that this approach could be readily standardized across multiple sites. Another advantage of this system is that accurate results could be obtained using only 3 ng DNA suggesting that the assay is well suited to applications with sample limitations (e.g., liquid biopsies, cell-free DNA). Furthermore, the ability to quantify low-abundance targets in the presence of complex genomic background suggests that this mdPCR system could be valuable for rare target detection, gene expression analysis, and quality control of complex samples.

This report provides proof-of-concept, but considerable work is required to determine the full potential of this approach. Further studies are required to investigate the numerous variables that could affect assay performance including sample characteristics (e.g., DNA quality and quantity), target sequences (e.g., SNPs vs. indels), probe design (e.g., melt determining regions), and detection systems (e.g., fluorescent reporters and quenchers). Another factor to consider is that mdPCR requires precise temperature control on a thermocycling platform that can image at several different set temperatures.

In summary, mdPCR shows excellent sensitivity, specificity, and reproducibility. Further optimization and validation will be necessary to realize the full potential of mdPCR, but this proof-of-concept study provides a strong foundation for future development and implementation of this approach. The versatility of this method makes mdPCR a promising tool for a variety of research and clinical applications. Importantly, this mdPCR system offers the opportunity for a new generation of dPCR with robust and high-order multiplexing that will make it possible to take advantage of the strengths of dPCR (absolute accuracy, precision, and rare event detection) for many applications that are impractical using current dPCR methods.

## Data Availability

The raw data supporting the conclusion of this article will be made available by the authors, without undue reservation.
