# Supplementary material for: Next generation multiplexing for digital PCR using a novel melt-based hairpin probe design
Source: Front Genet. 2023 Nov 10;14:1272964. doi: 10.3389/fgene.2023.1272964 (PMC10667681; doi:10.3389/fgene.2023.1272964)
Supplement: Supplementary file 1 [file Table1.DOCX]

Supplementary Material for

***Next generation multiplexing for digital PCR using a novel melt-based hairpin probe design***

Rebecca L. Edwards^1^, Johanna E. Takach^2^, Michael J. McAndrew^2^, Jondavid Menteer^3,4^,

Rachel M. Lestz^3,5^, Douglas Whitman^2^, Lee Ann Baxter-Lowe^1,3*^.

^1^Department of Pathology & Laboratory Medicine, Children’s Hospital Los Angeles, California, 90027, United States of America

^2^Luminex Corporation, A DiaSorin Company, Austin, Texas, 78727, United States of America

^3^Keck School of Medicine, University of Southern California, Los Angeles, California, 90033, United States of America

^4^Division of Cardiology, Children’s Hospital Los Angeles, Los Angeles, California, 90027, United States of America

^5^Division of Nephrology, Children’s Hospital Los Angeles, Los Angeles, California, 90027, United States of America.

Corresponding author: Lee Ann Baxter-Lowe

**Email:**  [lbaxterlowe@chla.usc.edu](mailto:lbaxterlowe@chla.usc.edu)

# Supplementary Data

Dataset S1 (separate file). Prototype assay oligonucleotide sequences.

**Dataset S2 (separate file).** Results of quantification of hge DNA mixtures (0.3% - 50%).

# Supplementary Tables

**Supplementary Table 1. Key features of targets and mdPCR design**

| **Assay** | **Locus** | **Chromosome** | **Allele** | **ID** | **Optical Color** | **Temperature Window** | **Amplicon Length** |
| --- | --- | --- | --- | --- | --- | --- | --- |
| P1 | rs34323650 | 22q11 | WT | A | Green | low | 110 |
|  |  |  | dup | B | Red | low | 112 |
| P2 | rs35629884 | 2q13 | WT | A | Green | mid | 93 |
|  |  |  | dup | B | Red | mid | 94 |
| P3 | rs10594381 | 8q24.1 | WT | A | Green | high | 100 |
|  |  |  | del | B | Red | high | 97 |
| P4 | rs3067729 | 20p11.2 | WT | A | Yellow | low | 74 |
|  |  |  | del | B | Blue | low | 72 |
| P5 | rs34669280 | 4p16 | WT | A | Yellow | mid | 95 |
|  |  |  | del | B | Blue | mid | 94 |
| P6 | rs34919970 |  | WT | A | Yellow | high | 109 |
|  |  |  | del | B | Blue | high | 108 |

dup, duplication; del, deletion; WT, wildtype.

**Supplementary Table 2. Comparison of multiplex digital PCR methods**

| **Method** | **Nucleic Acid Targets per assay** | **Technology Platform** | **Cycles** | **Target Identification** | **Limitations** |
| --- | --- | --- | --- | --- | --- |
| U-dHRM | Unbiased broad-based 16S rRNA gene primers | Custom U-dHRM chip | 70 | Generic reporter dye used to detect nucleic acids. Each molecule generates a specific melt profile. Identification by melt-curve matching to a database. | Potential for overlap of melt curves for different targets, requires a large database of temperature calibrated melt curves and has limited quantitative ability. |
| VPdPCR | 20 (10 targets per chromosome) | Droplet digital PCR | 45 | Varied concentration of TaqMan probes of the same color to identify of each probe using a distinct endpoint amplitude with in the same optical channel. | Complicated assay optimization, limited sensitivity (5% fetal/aneuploid fraction), and non-specific amplification. |
| BB-ddPCR | 5 viral targets | Droplet digital PCR | 70 | Pre-amplification the sample material, hybridizing the pre-amplified products to beads, bead partitioning, digital amplification the products and image analysis. | Lengthy multi-step workflow, imaging requires fluorescent microscopy. Demonstrated with only 5-plex assay. |
| mdPCR | 12 Indels | Absolute Q microfluidic dPCR (Software modified) | 49 | Each probe has a unique combination of fluorophore and melt window facilitating detection of each target in each partition. Images taken in four optical channels at temperatures, below and above probe Tm. | Requires a thermocycling platform capable of being held at specific temperatures during imaging. |
| BB-ddPCR, barcode bead-based multiplexed droplet digital PCR; Indels, Insertion and deletions; mdPCR, multiplex digital PCR; U-dHRM, Universal Digital High-resolution melt; VPdPCR, Virtual-partition digital PCR. | | | | | |
